# Effectiveness of reactive case detection for malaria elimination in three archetypical transmission settings: a modelling study

**DOI:** 10.1186/s12936-017-1903-z

**Published:** 2017-06-12

**Authors:** Jaline Gerardin, Caitlin A. Bever, Daniel Bridenbecker, Busiku Hamainza, Kafula Silumbe, John M. Miller, Thomas P. Eisele, Philip A. Eckhoff, Edward A. Wenger

**Affiliations:** 1 Institute for Disease Modeling, Bellevue, WA USA; 2 National Malaria Elimination Centre, Ministry of Health, Lusaka, Zambia; 3 PATH Malaria Control and Elimination Partnership in Africa, Lusaka, Zambia; 4 Center for Applied Malaria Research and Evaluation, Tulane University School of Public Health and Tropical Medicine, New Orleans, LA USA

**Keywords:** Malaria elimination, Reactive case detection, Stratification, Human movement, Mathematical modeling

## Abstract

**Background:**

Reactive case detection could be a powerful tool in malaria elimination, as it selectively targets transmission pockets. However, field operations have yet to demonstrate under which conditions, if any, reactive case detection is best poised to push a region to elimination. This study uses mathematical modelling to assess how baseline transmission intensity and local interconnectedness affect the impact of reactive activities in the context of other possible intervention packages.

**Methods:**

Communities in Southern Province, Zambia, where elimination operations are currently underway, were used as representatives of three archetypes of malaria transmission: low-transmission, high household density; high-transmission, low household density; and high-transmission, high household density. Transmission at the spatially-connected household level was simulated with a dynamical model of malaria transmission, and local variation in vectorial capacity and intervention coverage were parameterized according to data collected from the area. Various potential intervention packages were imposed on each of the archetypical settings and the resulting likelihoods of elimination by the end of 2020 were compared.

**Results:**

Simulations predict that success of elimination campaigns in both low- and high-transmission areas is strongly dependent on stemming the flow of imported infections, underscoring the need for regional-scale strategies capable of reducing transmission concurrently across many connected areas. In historically low-transmission areas, treatment of clinical malaria should form the cornerstone of elimination operations, as most malaria infections in these areas are symptomatic and onward transmission would be mitigated through health system strengthening; reactive case detection has minimal impact in these settings. In historically high-transmission areas, vector control and case management are crucial for limiting outbreak size, and the asymptomatic reservoir must be addressed through reactive case detection or mass drug campaigns.

**Conclusions:**

Reactive case detection is recommended only for settings where transmission has recently been reduced rather than all low-transmission settings. This is demonstrated in a modelling framework with strong out-of-sample accuracy across a range of transmission settings while including methodologies for understanding the most resource-effective allocations of health workers. This approach generalizes to providing a platform for planning rational scale-up of health systems based on locally-optimized impact according to simplified stratification.

**Electronic supplementary material:**

The online version of this article (doi:10.1186/s12936-017-1903-z) contains supplementary material, which is available to authorized users.

## Background

Malaria transmission is heterogeneous even at small scales, especially in low-transmission areas [1, 2]. Heterogeneity in proximity to vector breeding sites and coverage of interventions such as vector control and case management create local hotspots that can sustain transmission in an otherwise marginal setting [3–6]. As more regions reduce transmission, selective targeting of such transmission pockets becomes an attractive strategy for elimination. If effective, such targeting could save tremendous resources over an untargeted, blanket intervention strategy and potentially shorten a region’s time to elimination [2, 7].

Reactive case detection (RCD) is one such targeting strategy that has begun to be tested in the field and included in malaria control programme operations in several sub-Saharan African and southeast Asian countries [8–16]. During RCD, an index case of clinical malaria triggers follow-up activities in the household or neighbourhood of the index case; these follow-up activities differ widely and can include testing for fever or infection and treating those who test positive, presumptive treatment, or intensification of vector control. In theory, when transmission appears to be heterogeneous, individuals and vectors are poorly mixed, and malaria should spread slowly, making these settings good candidates for RCD [17]. However, field studies have not measured the effect of RCD on decreasing transmission, and pilot areas where RCD has been implemented have yet to achieve elimination [9, 12, 13]. It is ambiguous whether continued transmission is due to RCD requiring more time to show impact, insufficient coverage of RCD activities, or inherent properties of the study areas limiting the impact RCD can have.

What are the conditions under which RCD can be an effective strategy for elimination? Mathematical modelling is a useful platform for understanding malaria transmission across diverse settings within a unified framework and for comparing the effects of potential intervention options [18–20]. Spatial models can help elucidate the impact of interconnectedness and spatial heterogeneity in transmission and intervention coverage [21]. However, modelling RCD presents particular challenges as the relevant transmission and intervention scales are much smaller than are typically considered even with the finest-grained spatial models, and features such as the movement of infected vectors that can be ignored at larger scales must now be explicitly included.

In this work, the first dynamical models of *Plasmodium falciparum* malaria transmission at the household scale are presented. Three archetypical transmission settings are considered: low baseline transmission with clustered households; high baseline transmission with dispersed households; and high baseline transmission with clustered households.

**Fig. 1:**
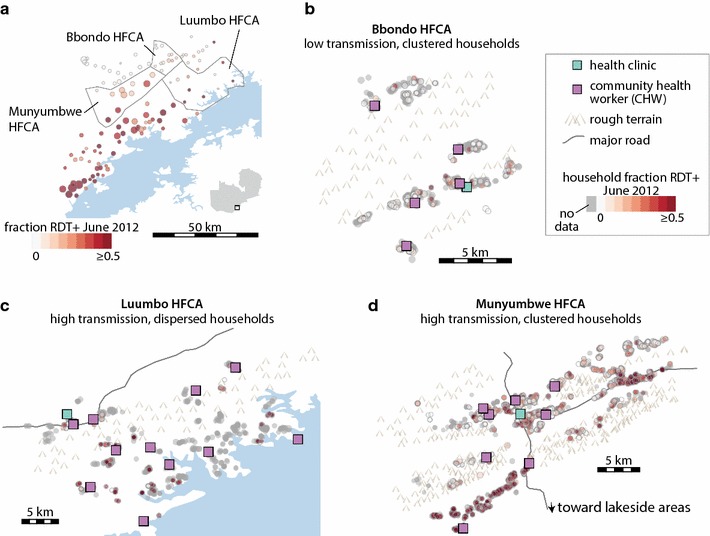
Three health facility catchment areas (HFCAs) in the Lake Kariba region of Southern Province, Zambia, span a range of transmission intensities and population densities. **a** Village-level prevalence of RDT-positive infections in June 2012 prior to mass drug campaigns shows higher transmission in lakeside areas and lower transmission in higher-altitude villages. Circle size is proportional to village population. **b** In Bbondo HFCA, households are highly clustered and baseline prevalence is low. **c** In Luumbo HFCA, households are dispersed and baseline prevalence is high. **d** In Munyumbwe HFCA, households are predominantly clustered around major roads, and baseline prevalence is mixed, with higher prevalence in the southwest valley and eastern roadside areas

**Fig. 2:**
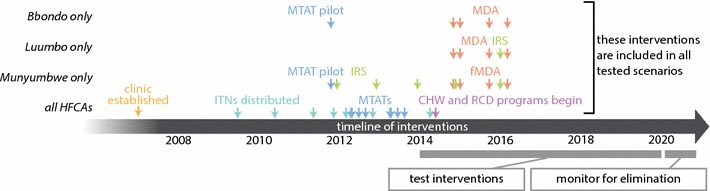
Timeline of interventions carried out in Bbondo, Luumbo, and Munyumbwe HFCAs from 2007 to 2016

To ground this study in realistic spatial heterogeneity in vectorial capacity and intervention coverage, health facility catchment areas (HFCAs) in the Lake Kariba region of Southern Province, Zambia, were used as model systems (Fig. 1). The Lake Kariba region spans transmission intensities from near-elimination to highly endemic and on par with some of the highest-transmission areas in sub-Saharan Africa [22]. Operational-scale studies of mass drug campaigns have been ongoing in this area since 2011, and RCD programmes began operation in mid-2014 (Fig. 2) [8, 23, 24]. The wealth of data collected at the household level in the Lake Kariba area enables parameterizing household-level models with fine-grained spatial and temporal patterns in transmission intensity and intervention coverage. These models can then be used to test and evaluate the effectiveness of RCD at interrupting transmission compared with other possible intervention strategies, including enhanced case management, vector control, mass drug campaigns, and limiting importations.

Answering the question of how to allocate scarce community health worker (CHW) time to labour-intensive RCD efforts compared to focusing on mass drug campaigns, case management of symptomatic malaria cases, or reduction of the burden of other diseases requires a clear understanding of malaria transmission at a household level and the spatial heterogeneities in transmission and connectivity. A simple stratification and classification of HFCAs would enable CHWs to be more-effectively placed and operationalized for achieving goals of malaria elimination and overall reduction of disease. To achieve such a stratification, the factors influencing CHW impact on actual malaria dynamics across the population and landscape must be measureable and simply-enumerated. Any model guiding such a stratification must also demonstrate out-of-sample accuracy across the range of applicable transmission settings across these enumerated factors.

## Methods

### Study site overview

In 2012–16, health facility catchment areas (HFCAs) in Gwembe District near Lake Kariba in Southern Province, Zambia, took part in operational-scale mass-test-and-treat (MTAT) [23] and mass drug administration (MDA) studies [24]. During these operations, data were collected on household locations, individual infection status as measured by rapid diagnostic test (RDT), recent symptoms and health-seeking behaviour, household ownership and individual usage of insecticide-treated nets (ITNs), and whether the household had recently received indoor residual spraying (IRS).

### Household model

All simulations were run with EMOD v2.10 [25], a stochastic agent-based model of malaria transmission that includes vector life cycle dynamics and within-host immunity effects [26, 27]. Relationships between force of infection, incidence, prevalence, and infectiousness have been calibrated to field data from study sites in sub-Saharan Africa [28]. Each household was modelled as a separate locus of transmission; within each household, transmission was homogeneous with biting propensities modulated by human size and individual ITN usage.

Household locations and sizes (Additional file 1) were constructed from a master list of households assembled from GPS locations and household rosters which included ages, genders, and household relationship structures recorded in the 2012–13 MTAT rounds data [23].

### Human movement

Each household had a randomly selected set of five other households within the HFCA that residents were permitted to visit, with each stay lasting an average of 3 days. For within-HFCA movement, each individual made on average 0.075 visits per year to other households. Migration rate within HFCAs was constant throughout the year.

In addition to moving between households within the same HFCA, individuals could also travel to an external high-transmission area that approximated lakeside regions. These lakeside trips lasted an average of 30 days. The external high-transmission area had a population of 1000 people, and residents of this area were not permitted to travel to the HFCAs. Because Munyumbwe HFCA is directly connected to lakeside areas by a major road, migration between Munyumbwe and the external high-transmission area was modelled at ten times the rate as for Bbondo and Luumbo HFCAs. In Bbondo, 95% of simulation-years had 38–65 individual trips to the external high-transmission area, with mean 0.0065 trips per person per year and 0.5–0.7% of Bbondo’s population visiting the external high-transmission area in any given year; in Luumbo, 95% of simulation-years had 67–97 trips, mean 0.0068 trips per person per year, and 0.5–0.8% of Luumbo’s population visiting the external high-transmission area in any given year; and in Munyumbwe, 95% of simulation years had 1569–1756 trips, mean 0.067 trips per person per year, and 6.3–6.9% of Munyumbwe’s population visiting the external high-transmission area in any given year. Following patterns seen in the MTAT rounds data in Munyumbwe HFCA, migration to the external high-transmission area was 10% higher between June and August. In Bbondo and Luumbo HFCAs, insufficient data was available to estimate relative seasonal migration rates, and migration rate to and from the external high-transmission area was modelled as constant throughout the year.

### Vector species and movement

Based on entomological studies conducted in the Lake Kariba region and other entomological data from Zambia ([29–31]; personal communication from Javan Chanda), both *Anopheles arabiensis* and *Anopheles funestus* vectors were included in the models. *Anopheles funestus* was parameterized with 95% indoor biting and *Anopheles arabiensis* with 50% indoor biting to account for outdoor resting behaviour, which limits the impact of ITN and IRS effect sizes [31]. These indoor biting fractions influence vector susceptibility to vector control interventions such as ITNs and IRS. *A. arabiensis* was modelled with rainfall-driven habitat peaking in February and a small amount of year-round available habitat, while *A. funestus* was modelled with a lagged rainfall-driven habitat component peaking in April and a dry season component peaking in November to reflect marshy areas that become fast-moving streams during the rainy season.

Vector numbers were determined by tuning the abundance of larval habitat associated with each household and modulated by seasonal patterns of rainfall; this process is described below in “Transmission intensity”. Each vector was modelled as an individual agent. Existing data on anopheline flight patterns is still sparse and highly dependent on experimental conditions and local geography [32], so a simple model of vector movement was implemented. Vectors migrated between households within an HFCA with distance-dependence as in the profile shown in Additional file 2, which was chosen based on literature review and calibration to mark-release-recapture studies [33, 34]. Vectors moved between households within 500 m apart at a high but decreasing rate depending on inter-household distance and could make trips of up to 3 km with a small probability; 90% of vector flights were within 370 m and 97% within 500 m. The distance-dependence of vector migration between pairs of households was weakly modulated by a propensity to migrate toward households with abundant associated larval habitat. Vectors did not migrate between HFCA households and the external high-transmission area. While local vector and human movement patterns were not explicitly varied in this study, the impact of spatial extent of transmission was measured indirectly by testing intervention mixes in areas of different household density.

### Intervention coverage

The timing and spatial coverage of ITN usage, IRS coverage, mass drug campaign coverage, and treatment-seeking rates were modelled according to self-reported data collected during mass treatment rounds (Additional files 1, 3, 4, 5, 6, 7).

### ITN parameterization

In simulation, ownership of an ITN implies nightly usage; in that way, ITN “coverage” and “usage” are interchangeable terms when referring to simulations. The fraction of people reporting having slept under a net last night during the drug campaign rounds was used to parameterize ITN coverage rates. To determine local ITN coverage, each HFCA was gridded at 1 km, ITN coverage was calculated for each grid cell, and household ITN coverage was modelled at the level seen over the grid cell (Additional files 3, 4, 5). As observed during campaign rounds, young children and adults were given a higher likelihood of receiving a net than school-age children [21]. At each MTAT or MDA/fMDA round, individuals were identified who reported sleeping under a net they received after the previous round of data collection. These nets were then distributed according to the reported net age and gridded coverage. ITN efficacy was parameterized with initial efficacy of blocking and killing of 0.9 and 0.6 and half-lives of 2.5 and 1.5 years, respectively for both vector species [35]. Based on net usage and ages reported across campaign rounds, individuals were modelled to discard ITNs after a mean retention time of 9 months. ITN distributions after 2016 were modelled as occurring in on June 15.

### IRS parameterization

Indoor residual spraying coverage was parameterized with a similar approach to ITNs. IRS timing was backdated from rounds data reporting on how long ago houses were sprayed. Campaigns after the Jan 2016 spraying were assumed to continue as planned and were modelled occurring in early January. Each household within a grid cell received IRS with probability equal to the fraction of households in that grid cell reporting having received IRS that year (Additional file 6). IRS was parameterized similarly for both vector species. Prior to 2015, IRS had initial killing efficacy of 0.5 and half-life of 2 months. Beginning with the Jan 2015 spray campaign, spraying was modelled with initial killing efficacy of 0.6 and half-life of 18 months to reflect the switch to Actellic, a longer-lasting insecticide; this parameterization results in 20–25% reduction in vector numbers in the subsequent year when 50% of households receive IRS. Since Bbondo HFCA consistently had little to no reported spraying, with at most 4.5% of households reporting recent spraying in any round, IRS campaigns were not modelled there.

### Mass drug campaigns

Mass test-and-treat was distributed in June, August, and October of each of 2012 and 2013, and a pilot round of MTAT in December 2011 was also included for Bbondo and Munyumbwe HFCAs. Drug campaign coverage achieved in each HFCA during the MTAT rounds was estimated from the fraction of longitudinally linked individuals found within that HFCA in subsequent campaign rounds, corrected by the estimated linkage efficiency [21]. During simulation, MTAT and MDA coverage is random at the individual level, not correlated between rounds or within households. In Bbondo and Munyumbwe HFCAs, MTAT coverage was modelled as 60%, except for the pilot round, which was set to 30 and 40% respectively for Bbondo and Munyumbwe. In Luumbo HFCA, MTAT coverage was modelled at 40%, except for the June 2012 round, which was modelled at 20%. During MTAT, all covered individuals who tested positive by RDT received a full course of artemether–lumefantrine (AL). RDT sensitivity was assumed to be 40 parasites per µL when administered by campaign staff.

For the 2014–2016 mass drug campaigns, coverage was assumed to be the same as the MTAT coverage: 60% for Bbondo, 40% for Luumbo, and 60% for Munyumbwe. Bbondo and Luumbo HFCAs received MDA, where all individuals received a full course of DHA–piperaquine (DP) regardless of infection status, and Munyumbwe HFCA received focal MDA (fMDA), where only individuals residing in a household with someone who tested positive by RDT received DP [24]. MDA or fMDA rounds were distributed in December 2014, February 2015, September 2015, and February 2016.

### Case management

Simulated case management rates prior to 2014 reflected campaign data collected during the 2012−2013 MTAT rounds, which showed both age- and distance-dependence of health-seeking behaviour [21]. Children under 15 years of age had a relative 50% greater chance of receiving care than adults. All clinical and severe cases that received treatment received an age-appropriate dose of AL within 3 days of presenting with symptoms.

Baseline case management rate modelled health-seeking behaviour depending on distance from the local clinic as observed during the MTAT rounds (Additional file 7). Case management rates after the CHW programme began in mid-2014 were assumed to increase as follows: cases in households within 1 km of the CHW’s location sought treatment at 60% for adult cases and 90% for child cases, and households beyond 1 km of a CHW followed the same distance-dependence as was observed for the clinics during the MTAT rounds. CHW and clinic locations were obtained from Zambia’s DHIS2 database [36].

### Reactive case detection

During RCD, programme guidelines indicate that a treated clinical case should trigger a CHW to test-and-treat all individuals in households within 140 m of the index case [8]. This was replicated in the model. Sensitivity of RDTs given during RCD was assumed to be 100 parasites per µL, a lower sensitivity than during MTAT as CHWs may have more difficulty with proper interpretation of RDT results than campaign staff [37], and all treatments used AL.

Since CHWs did not report which active follow-ups are triggered by which passive cases, it is difficult to ascertain the rate at which index cases were followed up with reactive activities and whether the follow-up procedure correctly followed the 140 m-radius guidelines. During follow-up activities, standard RCD was parameterized as 40% of index cases triggered follow-up, test-and-treat conducted within 140 m of index household, and 60% of people available for test-and-treat during follow-up [11]. Solely for out-of-sample comparisons in Munyumbwe HFCA, RCD was modelled as follows: in the northeast area where there is no CHW, no RCD was modelled. In the southwest region where transmission has historically been very high, 20% of index cases triggered follow-up, follow-up occurred only within the household of the index case, and 40% of people were available for test-and-treat. In the central region where the major roads intersect and many CHWs are present, 50% of index cases triggered follow-up, follow-up occurred within 50 m of the index household, and 40% of people were available for test-and-treat.

In hypothetical intervention scenarios, RCD was always modelled with a radius of 140 m from the index case. “Perfect” RCD indicates 100% of index cases occurring in the HFCA triggered follow-up activities and 100% of people were available for test-and-treat during follow-up. In all RCD scenarios, follow-up activities were modelled to occur the same day as treatment of the index case. RCD distributed with focal test-and-treat (fTAT) gave drugs only to individuals testing positive by RDT while RCD distributed with focal drug administration (fDA) gave drugs to all individuals found within the target radius. In all simulations, no upper limit on the number of people tested or treated with RCD was imposed.

### Transmission intensity

Household-associated larval habitat abundances and intervention coverages together determine each household’s baseline transmission intensity. Each household’s habitat abundance contributed to local vector numbers, household biting rate, and thereby individual risk of testing positive by RDT. Larval habitat abundances were selected from 200 sets of random household habitat abundances by comparing resulting simulation outputs to two types of data from the 2012–13 MTAT rounds: (1) HFCA-wide prevalence by RDT measured during the seven rounds of MTAT between 2011 and 2013, and (2) from the June 2012 data, the probability of an individual testing positive given another positive individual within the same household, within 50 m but not in the same household, and between 50 and 200 m away (Additional file 8). In addition to HFCA-wide prevalence by RDT, prevalence measurements for subregions in Luumbo and Munyumbwe were also considered as calibration targets as village-scale baseline prevalence in Luumbo and Munyumbwe showed considerable heterogeneity (Additional file 9).

Comparisons between simulations and observed data were calculated using euclidean distance, and distances for all comparisons were summed to calculate overall score. The 20 lowest-score habitat abundance samples were used for out-of-sample predictions and scenario projections. Data collected during the 2014–16 MDA and fMDA campaigns, routine clinical case counts from health facilities, and data collected by CHWs were not used for model calibration.

In Bbondo HFCA, all households had the same ratio of *arabiensis* and *funestus* habitat availability. In Luumbo and Munyumbwe, households within each subregion had the same ratio of *arabiensis* and *funestus* habitats; subregion-specific ratios were chosen based on preliminary entomological data in nearby sites to reflect subregion-specific seasonality.

### Scenario projections

For all HFCA models, all vector control (ITN and IRS) and mass drug distributions (MTAT, fMDA, MDA) interventions prior to 2017 were included in the intervention scenario packages (Fig. 2). Various potential case management rates, RCD coverages and methodologies, vector control distributions, additional mass drug campaigns, and malaria control measures implemented in the external high-transmission area were simulated over the period January 2014 through December 2021.

All intervention packages in the high-transmission area included annual IRS at 50% coverage and 30% case management rate. In addition, each intervention package included: (package 1) ITNs distributed every 3 years at 30% coverage; (package 2) ITNs every 3 years at 60% coverage; (package 3) ITNs every 3 years at 80% coverage; (package 4) ITNs every 2 years at 80% coverage; (package 5) ITNs every 2 years at 80% coverage and two MDA rounds each year at 70% coverage.

Each intervention scenario was simulated with 1000 stochastic realizations, 50 for each of the 20 best habitat abundance samples, to capture the range of possible outcomes. Realizations of each scenario where zero locally-acquired infections occurred over the entire 2020 calendar year were counted to have achieved elimination [38]. Locally-acquired infections included all new infections acquired in Bbondo, Luumbo, or Munyumbwe HFCA, including completely asymptomatic infections, but not infections acquired in the external high-transmission area and imported into Bbondo, Luumbo, or Munyumbwe.

## Results

### Household models of three archetypes of malaria transmission settings

Household-level models were constructed based on HFCAs in Southern Province, Zambia, to represent three archetypes of near-elimination settings (Fig. 1; Table 1). Bbondo HFCA is a remote area with historically lower transmission where households are clustered tightly into villages. Luumbo and Munyumbwe HFCAs are each larger than Bbondo in geographical area, and Munyumbwe contains over twice the population. In Munyumbwe, most households lie in the valley regions along two main roads connecting Munyumbwe to larger population centers in the east and lakeside areas that people visit for farming and fishing, while in Luumbo, households are more dispersed.

**Table 1:** Features of the Lake Kariba HFCAs chosen as model systems for three archetypical settings of malaria transmission

	Bbondo HFCA	Luumbo HFCA	Munyumbwe HFCA
Number of households	745	744	3152
Population	8000	12,000	25,000
Approximate total area (km^2^)	118	461	340
Occupancy area	0.18	0.38	0.16
RDT+ prevalence in June 2012	9%	42%	28%

Total area of each HFCA was approximated by a concave hull with α = 0.1 around household locations. Occupancy area, a measure of household clustering, was calculated by drawing a circle of 200 m around each household, calculating the total area covered by at least one circle, and normalizing by the maximum occupancy area achieved if no circles overlap. The 200 m buffer was chosen to reflect a realistic daily mosquito flight distance. Lower occupancy area indicates more clustered households

Many interventions have been distributed in these areas over the last decade (Fig. 2), although coverage was often quite patchy. Based on household respondent self-reporting, parts of these HFCAs were consistently underserved by ITN distributions (Additional files 3, 4, 5). Households close to major roads were most likely to receive IRS in both Luumbo and Munyumbwe HFCAs despite less accessible areas often having higher burden (Additional file 6). Even after the introduction of CHWs, a number of households in northeast Bbondo, eastern Luumbo, and eastern and southwestern Munyumbwe remained far from sources of treatment and may continue to experience insufficient access to care, and any RCD is less likely to perform at programme targets in those areas (Additional file 7).

**Fig. 3:**
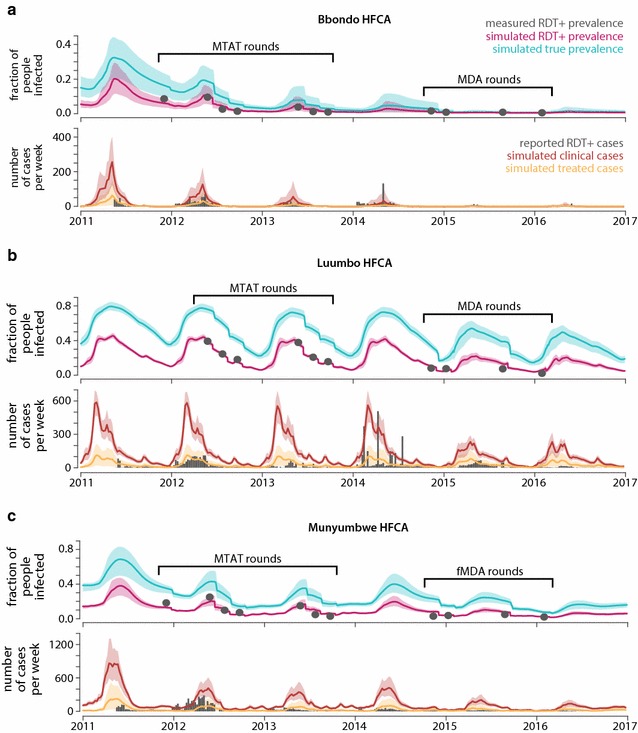
Simulated transmission intensity in **a** Bbondo **b** Luumbo and **c** Munyumbwe HFCAs compared with RDT prevalence measurements taken during MTAT and MDA/fMDA rounds and RDT-confirmed fevers reported by health facilities and CHWs. In the *bottom row* of each *panel*, simulated clinical cases included both treated and untreated symptomatic cases of malaria, while simulated treated cases are cases that have contacted the health system and would potentially show up in clinic and CHW reporting. The relevant comparison is therefore between the *gray bars* and the *yellow line* and *area*. Each simulation trace shows the mean and range of 1000 realizations, with the *yellow shaded area* expanded by 150% to indicate additional uncertainty in treatment and reporting rates

The household models, which were calibrated using the 2012–13 MTAT data on prevalence of RDT+ infections, also captured prevalence by RDT collected during the 2014–16 fMDA/MDA rounds and the number of RDT-positive fevers reported at health facilities and by CHWs (Fig. 3). Agreement with the fMDA/MDA round out-of-sample observations suggests that the models were reasonable representations of local transmission dynamics and intervention effect sizes: root mean square error between simulation and field measurements over the fMDA/MDA rounds was 0.0021, 0.039, and 0.030 for Bbondo, Luumbo, and Munyumbwe HFCAs respectively.

While the models broadly captured observed patterns in reported clinical cases, a variety of factors, including year-to-year variation in climate, seasonal variation in health-seeking behaviour due to accessibility and perceptions of whether a fever is malarial, and inconsistent record-keeping make discrepancies between model and surveillance difficult to interpret. Forward projections shown in subsequent sections should therefore be taken as representative outcomes in archetypical transmission settings rather than predictions of how transmission will evolve in these specific areas over the next few years. Furthermore, although Luumbo and Munyumbwe HFCAs had high prevalence at baseline, current transmission intensity is much lower, and this context of recently reduced transmission should be kept in mind when interpreting outcomes of potential intervention scenarios.

### In areas with historically low transmission, path to elimination includes limiting importation and increasing case management rate

**Fig. 4:**
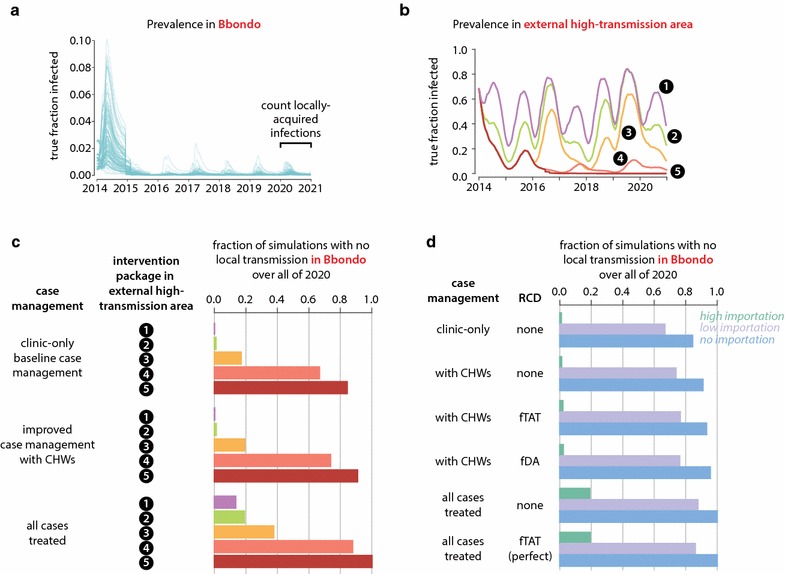
Success of elimination programmes in Bbondo HFCA is dominated by effects of reducing importation and improving case management. **a** Transmission in Bbondo HFCA was monitored under 1000 realizations of each intervention scenario from 2014 through 2020. Simulations with zero locally-acquired infections in 2020 were considered to have achieved elimination. **b** Five potential intervention packages were modelled in the external high-transmission area to which Bbondo residents may travel. See “Methods” for details on these intervention packages. **c** Probability of elimination in Bbondo under three potential case management rates and five potential intervention packages implemented in the external high-transmission area. **d** Probability of elimination in Bbondo under various case management rates and potential RCD implementations. Perfect RCD indicates 100% of index cases receiving follow-up and 100% of residents at home and receptive to follow-up activities. Scenarios were simulated under three importation rates, corresponding to intervention package #5 in the external high-transmission area (no importations), package #4 (low importation), and package #2 (high importation)

Transmission in Bbondo HFCA was projected forward under a variety of intervention scenarios (Fig. 4; Additional file 10). For each scenario, transmission was monitored through the end of 2020, and simulations where no infections were acquired in Bbondo over the entire 2020 year were counted to have achieved elimination (Fig. 4a).

In addition to interventions modelled in Bbondo, five potential intervention packages were considered for the external high-transmission area to account for malaria control or elimination operations that happen in tandem across a wider region (Fig. 4b). These intervention packages resulted in a range of transmission intensities in the external high-transmission area, from sustained high transmission to elimination, and thus modulated the rate of infection importation into Bbondo (Additional file 11).

Simulations predicted that decreasing importation rate into a low-transmission area like Bbondo HFCA could result in elimination by the end of 2020 even when case management was the only malaria control intervention operating in Bbondo after the 2014 ITN distribution and 2014–16 MDA rounds, and RCD was not implemented (Fig. 4c). Stopping importation by eliminating in the external high-transmission area led to elimination in Bbondo in 84% of simulations where case management occurred at the low levels observed prior to the CHW programme. If all cases in Bbondo were to be treated, simulations predicted 100% chance of elimination by the end of 2020. As long as rigorous control operations are ongoing in connected high-transmission areas, there is little need for interventions beyond case management to be implemented in low-transmission areas, and increasing case management rate though community-based case management increases the probability of achieving elimination at all importation rates tested.

Conversely, when case management in Bbondo was greatly improved but importations continued and no other interventions were occurring in Bbondo, elimination was possible but unlikely, with only 14% of simulations eliminating in scenarios with the highest importation rate. Travellers who return from the external high-transmission area with untreated infections can still transmit to Bbondo residents, although few, if any, secondary cases will arise when case management rates are high. Implementing RCD in Bbondo increased probability of elimination only to 17%, even if RCD effectiveness was maximized by assuming perfect coverage (Fig. 4d). Changing RCD methodology from focal MTAT to focal MDA had minimal effect for all case management and importation rates, suggesting that parasite-positive RDT-negative individuals are not contributing substantially to transmission in Bbondo and that prophylactic benefits from presumptive treatment are small. Additional mass ITN distributions in Bbondo increased probabilities of elimination by only an average of 1.5 percentage points (Additional file 10).

### Elimination in dispersed, high-transmission areas requires limiting importations, improving case management, maintaining vector control, and depleting the asymptomatic reservoir

**Fig. 5:**
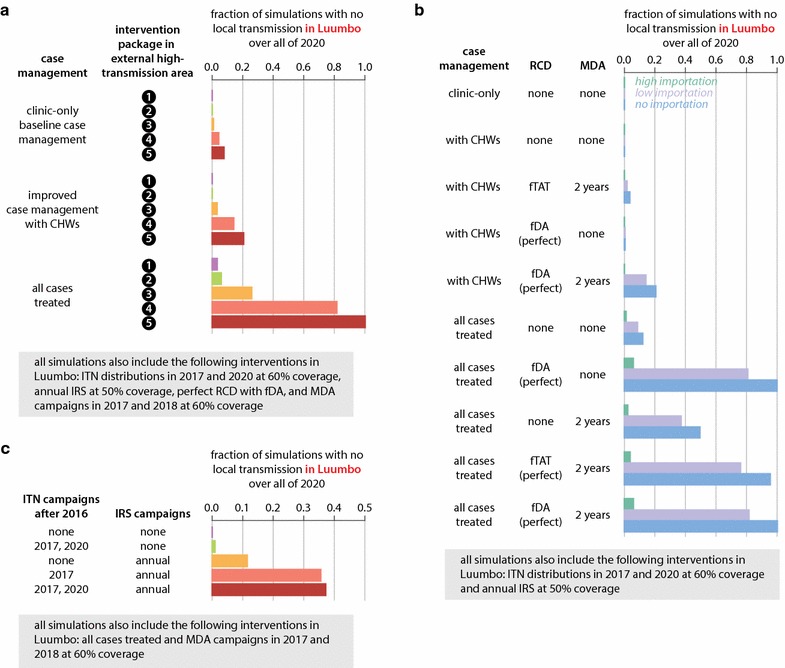
Elimination in Luumbo HFCA requires limiting importations, increasing case management rate, maintaining vector control, and either RCD or MDA campaigns. **a** Probability of elimination in Luumbo under three potential case management rates and five potential intervention packages implemented in the external high-transmission area. **b** Probability of elimination in Luumbo under various case management rates and potential RCD implementations. Importation rates correspond to intervention packages in the external high-transmission area as described in the caption to Fig. 4d. **c** Probability of elimination in Luumbo under various vector control packages implemented in Luumbo. All intervention scenarios were simulated under low importation (intervention package #4 in external high-transmission area)

In sparsely-populated high-transmission areas such as Luumbo HFCA, elimination is possible and even likely in certain circumstances (Fig. 5; Additional file 10). Under a comprehensive and well-implemented elimination programme that included RCD, MDA, and regular refreshing of ITNs and IRS, simulations predicted that elimination in Luumbo was very likely as long as case management rates were excellent and importations were stopped or severely limited (Fig. 5a).

Limiting importations and improving case management without including other interventions is unlikely to be sufficient for elimination in areas like Luumbo HFCA (Fig. 5b; Additional file 10). RCD and MDA increased the chances of elimination if well-implemented. RCD with focal MDA resulted in slightly better outcomes than RCD with focal MTAT, as subpatent infections are more likely to be transmitted when vectorial capacity is high. In Luumbo, excellent case management was a requirement for elimination, and transmission was interrupted in only 21% of simulations with estimated CHW-based case management rates, even if both RCD and MDA were also part of programme operations and importations were stopped. Under the current strategy of focal MTAT and MDA campaigns, simulations predicted elimination in Luumbo to be unlikely even if importations from connected high-transmission areas ceased (Fig. 5b, third row).

Maintaining good coverage with vector control was a critical component of successful elimination operations in Luumbo (Fig. 5c). In simulations, both ITN distributions and IRS campaigns increased the likelihood of elimination, particularly when deployed together, when other prerequisites for elimination were met. Vector control alone at the levels simulated was insufficient for elimination in Luumbo.

### Elimination in clustered, high-transmission areas requires high-quality implementation of all existing tools

**Fig. 6:**
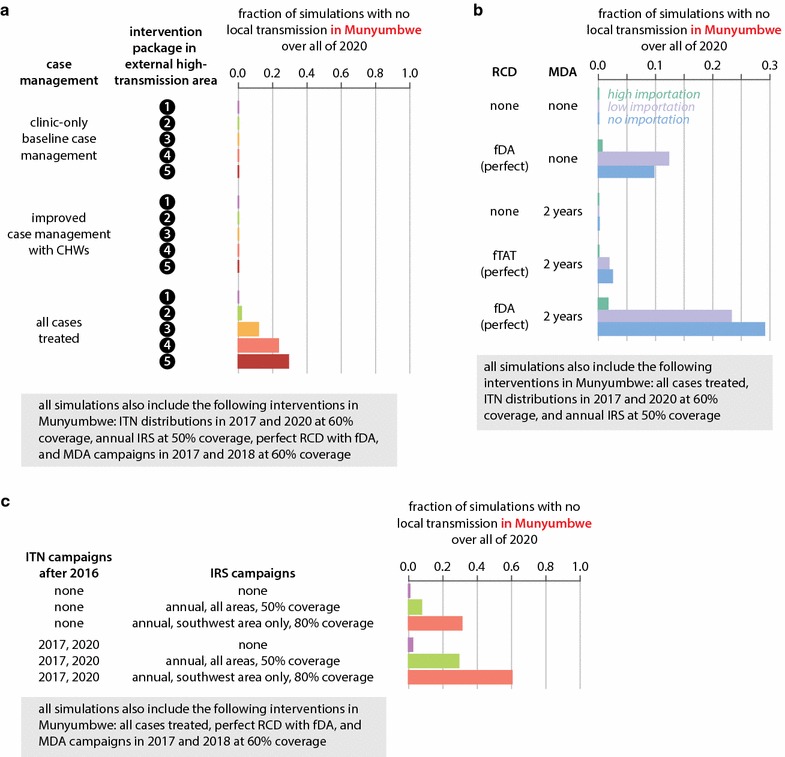
Elimination in Munyumbwe HFCA requires limiting importations, increasing case management rate, enhancing vector control with targeted high-coverage campaigns, and both RCD and MDA. **a** Probability of elimination in Munyumbwe under three potential case management rates and five potential intervention packages implemented in the external high-transmission area. **b** Probability of elimination in Munyumbwe under various case management rates and potential RCD implementations. Importation rates correspond to intervention packages in the external high-transmission area as described in the caption to Fig. 4d. **c** Probability of elimination in Munyumbwe under various vector control packages implemented in Munyumbwe. All intervention scenarios were simulated under no importation (intervention package #5 in external high-transmission area)

In highly clustered high-transmission areas, any infection is potentially transmissible to a large number of people, making elimination particularly challenging. Limiting imported infections, excellent case management, maintenance of vector control, and depleting the asymptomatic reservoir with both RCD and MDA were necessary for elimination in simulations of Munyumbwe HFCA (Fig. 6; Additional file 10). However, even under these circumstances, only 29% of simulations achieved elimination (Fig. 6a).

As in the other transmission archetypes discussed above, case management was a crucial and necessary component of elimination strategies in Munyumbwe HFCA. Even when vector control, MDAs, and interventions limiting importations were in place, leaving too many symptomatic cases untreated prevented successful interruption of transmission. In dispersed high-transmission areas, excellent case management alone could occasionally interrupt transmission without the help of RCD or MDA (Fig. 5b, sixth row), and adding an MDA programme on top of excellent case management and RCD offered little benefit (Fig. 5b, bottom row). In contrast, in clustered high-transmission areas, case management without RCD or MDA was never observed to lead to elimination within the timeframe under consideration (Fig. 6b, top row), and including both MDA and RCD in elimination operations resulted in more likely elimination than either drug-based strategy alone (compare Fig. 6b second, third, and bottom rows). Prophylactic benefits from presumptive treatment are especially powerful in high-transmission areas where risk of infection is high [39, 40]. As in Luumbo, focal MDA was a superior response strategy to focal MTAT in RCD as subpatent infections are more likely to be transmitted when the infected individual is subjected to more biting.

As expected, vector control was also a critical component of any elimination programme in Munyumbwe HFCA. When coverage with ITNs or IRS is suboptimal, as is often the case due to a variety of factors from resource limitation to logistical challenges with delivery and individual behaviour, combining multiple forms of vector control can be particularly beneficial (Fig. 6c).

Taking advantage of known local heterogeneities in transmission, if any, can improve outcomes by more efficient targeting of resources. In Munyumbwe HFCA, the southwestern valley has historically had more malaria than other regions (Fig. 1d; Additional file 9). By reconfiguring annual IRS campaigns, which were simulated at 50% coverage evenly across Munyumbwe, to specifically target households in the southwest area at 80% coverage while forgoing IRS in other parts of Munyumbwe, 60% of simulations now achieved elimination by the end of 2020 compared with only 29% with untargeted IRS (Fig. 6c). If vectorial capacity is uniformly high, widespread enhanced vector control will be necessary.

### Composition of the infectious reservoir determines whether targeting asymptomatic infections is necessary for rapid elimination

**Fig. 7:**
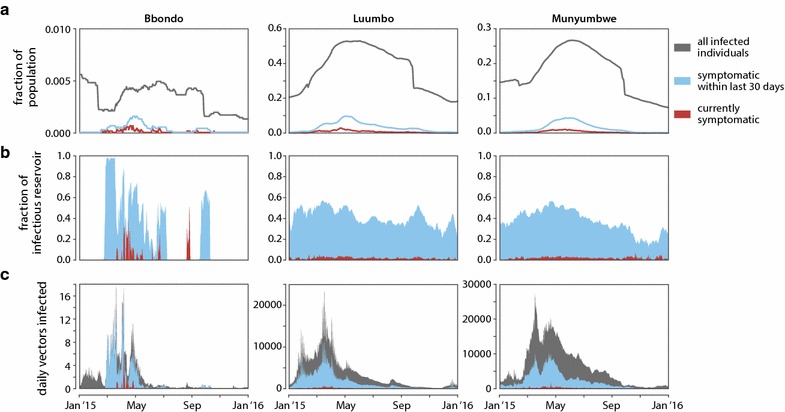
Contribution of recently symptomatic individuals to the infectious reservoir in Bbondo, Luumbo, and Munyumbwe HFCAs. For each HFCA, a representative simulation was run over the year 2015 at baseline case management rate with no RCD but including any vector control and MDA or fMDA campaigns. The external high-transmission area received intervention package #1. **a** Prevalence of infected individuals, currently symptomatic individuals, and individuals who were symptomatic within the last 30 days but not currently symptomatic. **b** Relative contribution to the infectious reservoir in each HFCA from current and recent symptomatics. **c** Daily number of vectors infected by currently symptomatic, recently symptomatic, and all other individuals in each HFCA

While individuals who are currently symptomatic make up only a tiny portion of the infectious reservoir at all levels of transmission [41], untreated individuals who have recently experienced symptoms form a substantial portion of the reservoir, particularly during the transmission season in low-transmission areas (Fig. 7). Human infectiousness is highly dependent on gametocyte density, which is driven by asexual parasite density [42]. Since asexual density is highest toward the beginning of an infection [43], individuals who were recently symptomatic are disproportionately more infectious than those who were not recently symptomatic and therefore contribute to a greater share of the infectious reservoir than their prevalence in the population would suggest.

In Bbondo HFCA, simulations showed that during the transmission season, few vectors were being infected by individuals who were not recently symptomatic (Fig. 7c). Thus, increasing case management in low-transmission areas potentially eliminated the bulk of onward infection, and every transmission season was another opportunity to interrupt transmission with case management. Additional measures such as RCD that targeted asymptomatic individuals therefore offered little additional benefit in low-transmission areas.

In historically high-transmission areas such as Luumbo and Munyumbwe HFCA, recently symptomatic individuals made up at most 50% of the infectious reservoir (Fig. 7b). Asymptomatic individuals contributed substantially to onward transmission, and even if all symptomatic cases were to be treated, simulations expected enough vectors to continue to be infected that transmission would not be interrupted. In high-transmission HFCAs, simulations predicted that a drug-based intervention such as RCD or MDA capable of targeting this asymptomatic reservoir would be necessary to achieve elimination.

## Discussion

In all three transmission settings considered in this study, reducing importations from connected high-transmission areas was a critical component of elimination success. In the low-transmission setting, reducing importations was the single most impactful intervention, as once importations stop, elimination can be achieved even when many cases are left untreated. In high-transmission settings, reducing importations is a prerequisite for elimination, as even a highly aggressive combination of local interventions fails to sustain interruption in transmission if imported infections continue. While not examined in this study, the seasonality of human movement can intersect with seasonality of vector numbers in ways that amplify the effects of importation or alter the seasonal profile of transmission. For example, temporary migration to lakeside areas during the dry season for fishing would result in importing infections and jump-starting transmission at the beginning of the wet season in areas further from the lake. Imported infections can be addressed through screening and treatment of travellers returning from high-transmission areas, a challenging prospect even at many international borders much less domestically, or through intensification of control interventions in highly-connected high-transmission areas. Regional approaches to malaria elimination are therefore necessary, and smaller interconnected areas cannot be considered completely separately; rather, resources spent on reducing burden in high-transmission areas will provide great benefits beyond those areas.

**Fig. 8:**
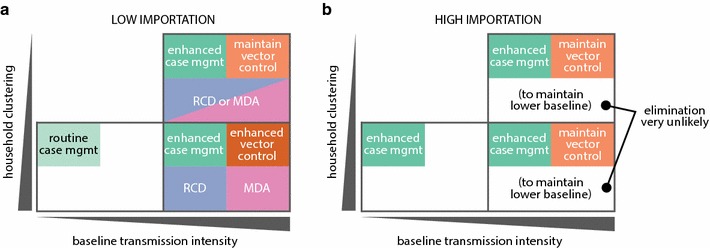
Recommended intervention mixes for elimination in **a** low- and **b** high-importation settings, stratified by local baseline transmission intensity and household clustering. High-clustering, low baseline transmission based on results from Bbondo HFCA; low-clustering, high baseline transmission based on Luumbo HFCA; high-clustering, high baseline transmission based on Munyumbwe HFCA

At the local level, good case management is a critical component of any elimination campaign (Fig. 8). A 22-year longitudinal study of a very high transmission area in Senegal saw prevalence and incidence decrease to near-zero levels with vector control and excellent case management [44]. When programmes wish to accelerate elimination in high-transmission areas, drug-based methods such as MDA and RCD that target the asymptomatic reservoir are also necessary.

Lingering immunity to clinical symptoms in areas with historically high transmission will influence how effective case management can be as an elimination tool. If immunity wanes slowly, targeting the asymptomatic reservoir is crucial; if immunity wanes rapidly, excellent case management becomes all the more important as the asymptomatic reservoir is present only seasonally rather than year-round. Unfortunately little is known about how long immunity is sustained as transmission declines [45], and misspecification of these effects could substantially impact modelled outcomes.

Low-transmission settings with dispersed households were not included in this study. Malaria risk in rural areas increases with population density [46], and malaria is more difficult to sustain in sparsely populated low-transmission areas than densely-populated low-transmission areas. Low-transmission low-density settings are inherently more marginal transmission environments than the other archetypes examined in this study, as small effective population sizes and low vectorial capacity limit the spread of infection. Case management alone is, therefore, also likely to be sufficient for elimination in lower-density low-transmission areas, provided that transmission is indeed primarily occurring in households. However, providing good access to treatment to low-burden highly dispersed populations remains operationally challenging.

Community-based case management is a widely-used method for increasing case management rates in resource-limited settings. CHWs tend to adhere to treatment guidelines for clinical malaria when conducting case management activities, but the extent to which RCD is implemented often varies substantially [11, 14, 47, 48]. Interruptions in supply chains for RDTs and drugs, insufficient CHWs for adequate coverage especially during the transmission season, inaccessibility of certain areas due to flooding or lack of infrastructure, and community members’ preference to seek treatment at health clinics rather than from CHWs all impact CHW performance in both their case management and RCD activities [48].

While this analysis predicts RCD has little benefit with respect to elimination outcomes in low-transmission areas, conducting RCD activities may have positive effects beyond clearing parasites from individuals living near index cases. RCD can increase CHW visibility in the community and could encourage CHWs to treat milder symptomatic cases, thereby increasing case management rate. RCD can help programmes improve mapping of households, which would be useful in any MDA or door-to-door vector control distributions. Test-and-treat RCD is an invaluable source of ongoing surveillance data on local prevalence of infection at a fine-grained spatial scale, potentially identifying areas for targeted vector control. Conducting RCD can also keep CHWs engaged in the elimination process and encourage continued high performance in finding and treating symptomatic cases.

In dispersed, high-transmission areas when RCD is well-implemented, simulations predict little contribution from MDA toward increasing the likelihood of elimination. However, MDA reduces burden on CHWs, improving their ability to conduct case investigations, and a well-timed MDA could allow CHWs to interrupt transmission over the subsequent season. In areas where CHWs cannot regularly operate due to inaccessibility, MDA may very well be the best way to clear the asymptomatic reservoir. In higher-transmission areas, perfect RCD is equivalent to frequent and well-targeted MDA, and monthly MDA during the transmission season at 60% coverage could show similar performance to a very well-implemented RCD program.

While current guidelines suggest implementing RCD in low-transmission areas because low-transmission areas are the most heterogeneous and case counts are low enough to make routine case investigation feasible [49], this study finds that RCD is most impactful toward accelerating elimination in areas with historically higher transmission that have recently seen decreases in transmission intensity. History matters when considering the interventions necessary to achieve elimination in an area with low prevalence. If transmission was historically low, that is, prevalence was low prior to recent intensification of control efforts, elimination will be easier and fewer interventions are required than if transmission was only recently decreased due to intensification of malaria control. Exactly how local vectorial capacity, heterogeneity of exposure, nature and timing of interventions, and duration of immunity come together to define whether an area is high- or low-transmission, and where the boundary between high and low lies, remain open questions.

When planning elimination operations, it is critical to understand an area’s transmission intensity over many years prior to the present. In areas without detailed records on transmission intensity, serology can be used to measure historical exposure [50]. Even if a region is not currently ready for elimination operations, improving surveillance and record-keeping will guide future programmes on where targeted intensification of vector control should be applied, where CHWs should be placed to improve case management, and whether targeting the asymptomatic reservoir should be included in elimination strategies.

Other archetypes of malaria transmission also exist in addition to the three examined here. Transmission can occur primarily through occupational exposure rather than at the household level, vector biting preferences will affect how effective standard vector control interventions can be, and species-specific vector dispersion behaviour will affect the optimal radius of RCD. Geography-specific virulence and infectiousness of parasite strains and differential human immune responses may further complicate how effective case management is as an elimination tool. Further work will be necessary to understand what intervention mixes would most efficiently lead to elimination in these other types of settings.

## Conclusion

The present approach can be extended to understanding effective health system resource-allocation in malaria-endemic settings more generally. Health facility capacity and community health workers are two essential resources of a primary health system, and constraints on these resources require that geographic and demographically targeted scale-up of each be guided by locally-achievable impact. It is not only important to be able to target scale-up of CHW resources according to a simple stratification based on easily-measureable factors, it is also necessary to prioritize activities and time-allocation for CHWs in each local area based on impact, especially for resource-intensive activities such as RCD. By focusing allocation of resources and time on those activities that have the strongest local impact, the overall resources available for malaria elimination and primary health care in general can go further.

## Additional files

**Additional file 1.** Household locations, populations, and intervention coverages. Household coordinates have been anonymized to kilometers from HFCA centroid to preserve participants’ privacy.

**Additional file 2.** Vector migration in household models.

**Additional file 3.** Observed ITN coverage in Bbondo HFCA between 2012 and 2015.

**Additional file 4.** Observed ITN coverage in Luumbo HFCA between 2012 and 2015.

**Additional file 5.** Observed ITN coverage in Munyumbwe HFCA between 2012 and 2015.

**Additional file 6.** Observed IRS coverage between 2012 and 2015 in Luumbo and Munyumbwe HFCAs.

**Additional file 7.** Modelled spatial variation in case management rate under baseline conditions and after implementation of the CHW programme.

**Additional file 8.** Conditional probabilities of testing positive by RDT in June 2012 given an RDT+ individual within the same household, given an RDT+ individual living within 50m but not in the same household, given an RDT+ individual living between 50m and 200m away, and overall prevalence in the HFCA.

**Additional file 9.** Luumbo and Munyumbwe HFCAs show village-scale differences in baseline prevalence.

**Additional file 10.** Table of elimination outcomes for all simulated scenarios.

**Additional file 11.** Importation rates into HFCAs under four potential intervention packages in the external high-transmission area.

## Abbreviations

AL: artemether–lumefantrine
CHW: community health worker
DP: dihydroartemisinin–piperaquine
fDA: focal drug administration
fMDA: focal mass drug administration
fTAT: focal test-and-treat
HFCA: health facility catchment area
IRS: indoor residual spraying
ITN: insecticide-treated net
MDA: mass drug administration
MTAT: mass test-and-treat
RCD: reactive case detection
RDT: rapid diagnostic test
