# Supplementary figures and images for: Effectiveness of reactive case detection for malaria elimination in three archetypical transmission settings: a modelling study
Source: Malar J. 2017 Jun 12;16:248. doi: 10.1186/s12936-017-1903-z (PMC5469005; doi:10.1186/s12936-017-1903-z)

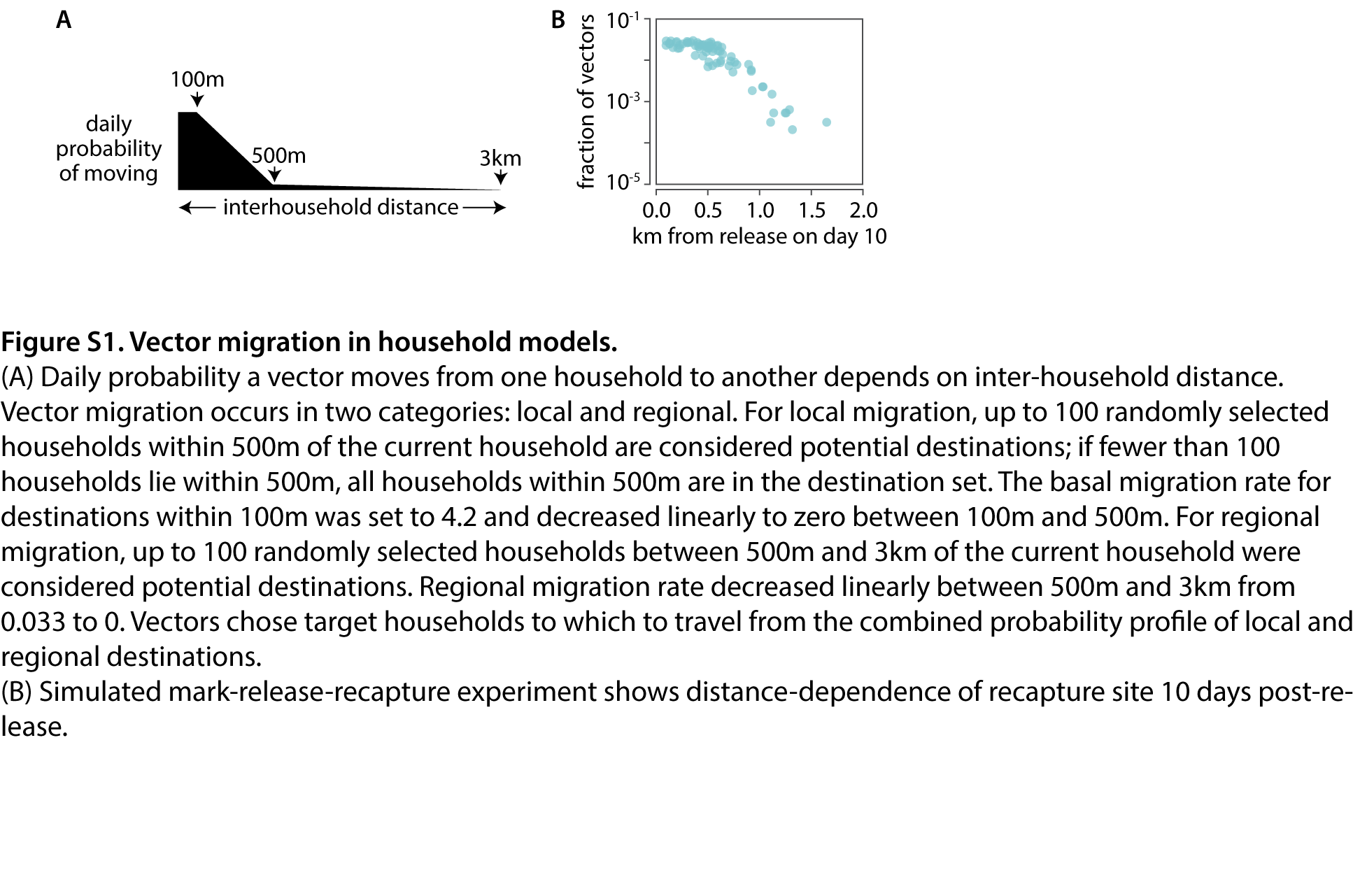

Supplement: Supplementary file 2 — Additional file 2. Vector migration in household models. [file 12936_2017_1903_MOESM2_ESM.png]

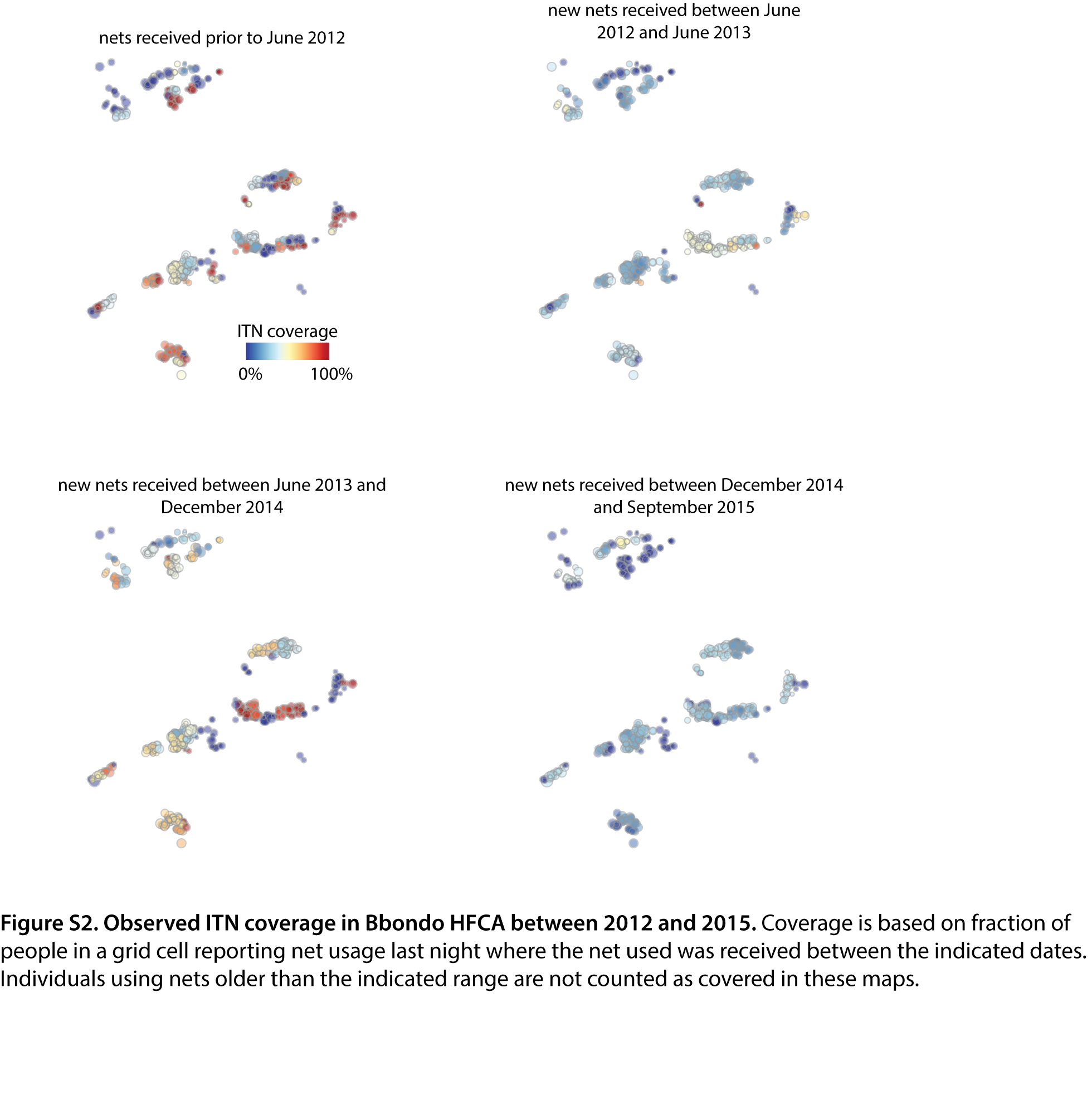

Supplement: Supplementary file 3 — Additional file 3. Observed ITN coverage in Bbondo HFCA between 2012 and 2015. [file 12936_2017_1903_MOESM3_ESM.png]

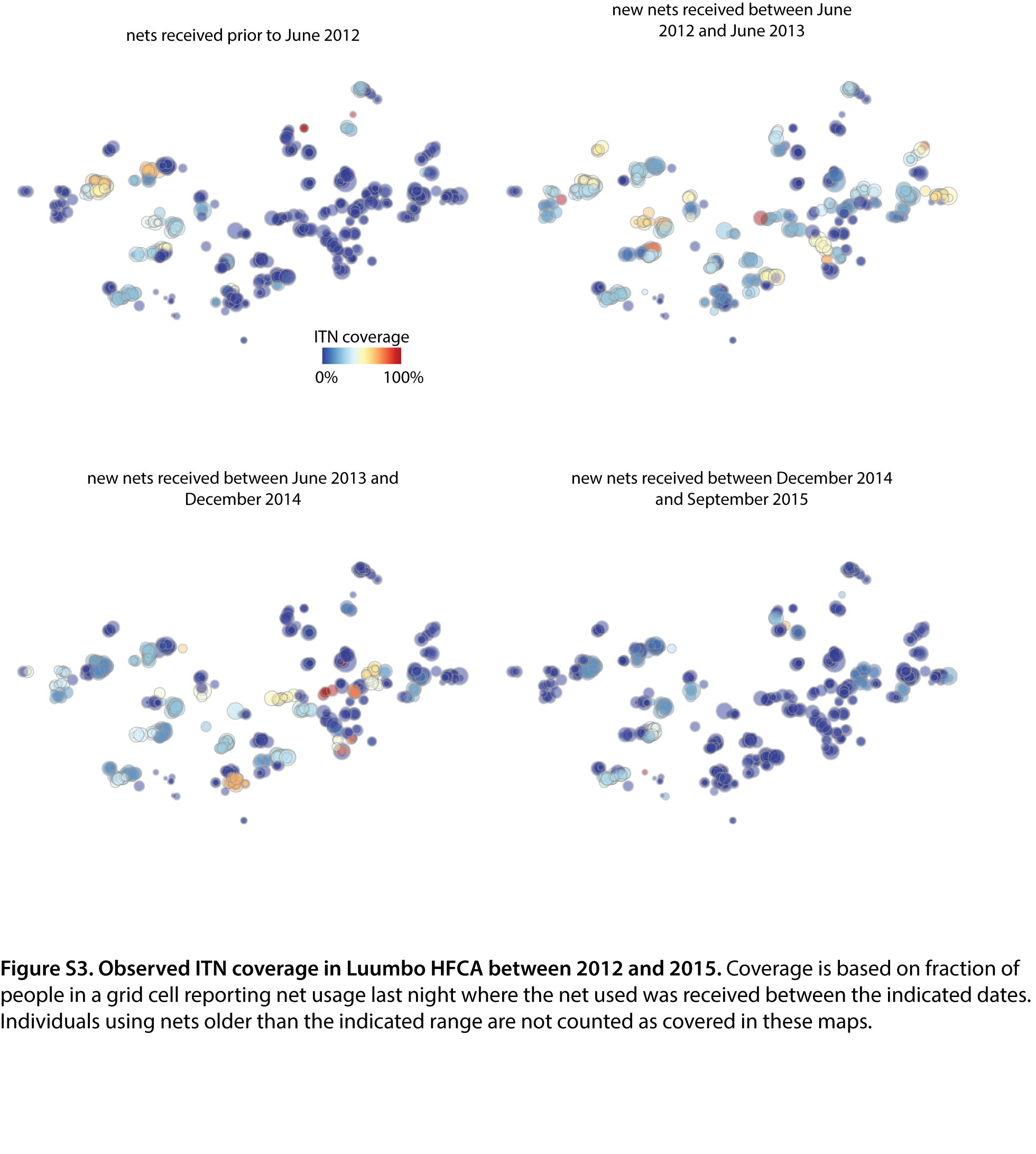

Supplement: Supplementary file 4 — Additional file 4. Observed ITN coverage in Luumbo HFCA between 2012 and 2015. [file 12936_2017_1903_MOESM4_ESM.png]

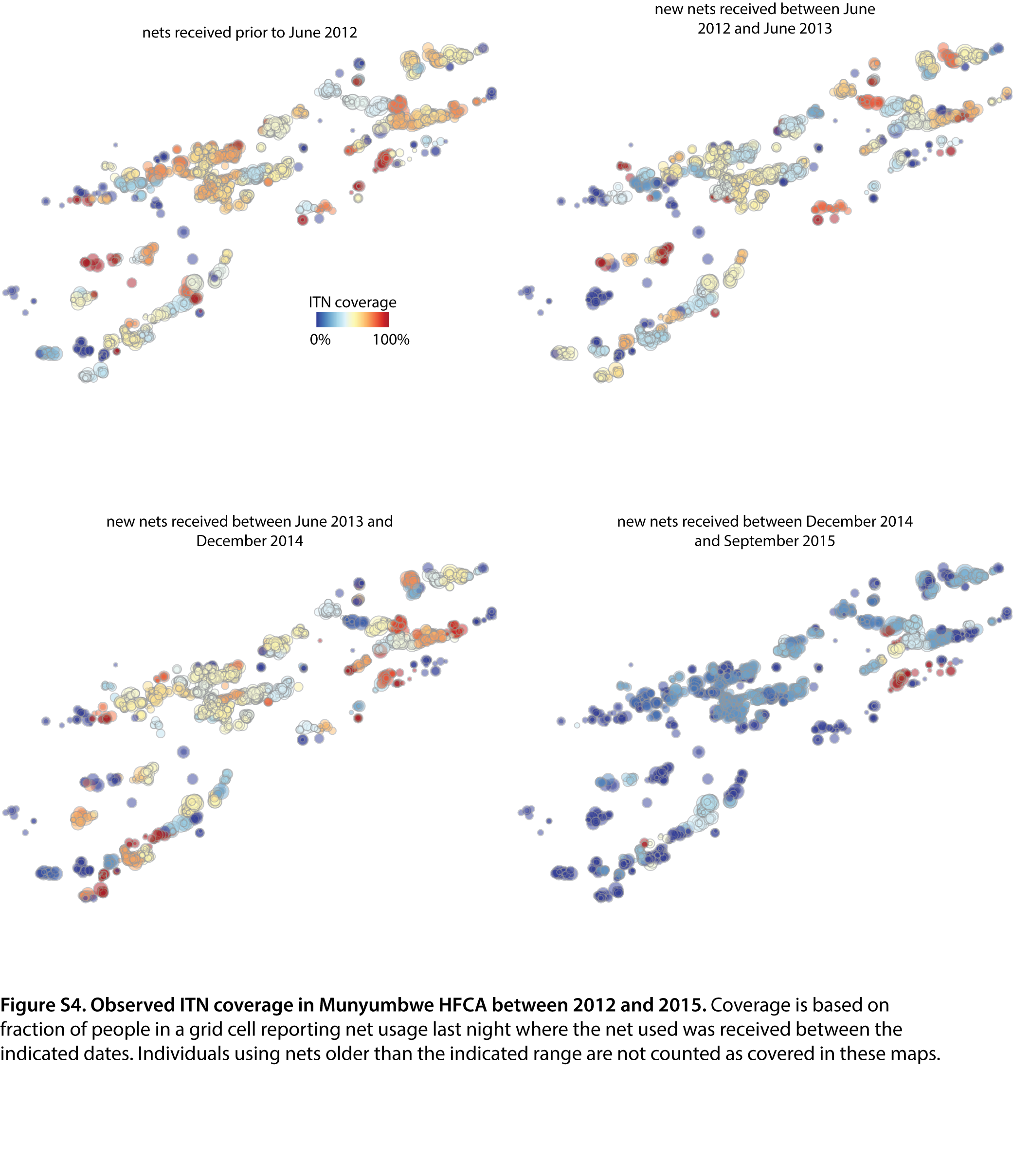

Supplement: Supplementary file 5 — Additional file 5. Observed ITN coverage in Munyumbwe HFCA between 2012 and 2015. [file 12936_2017_1903_MOESM5_ESM.png]

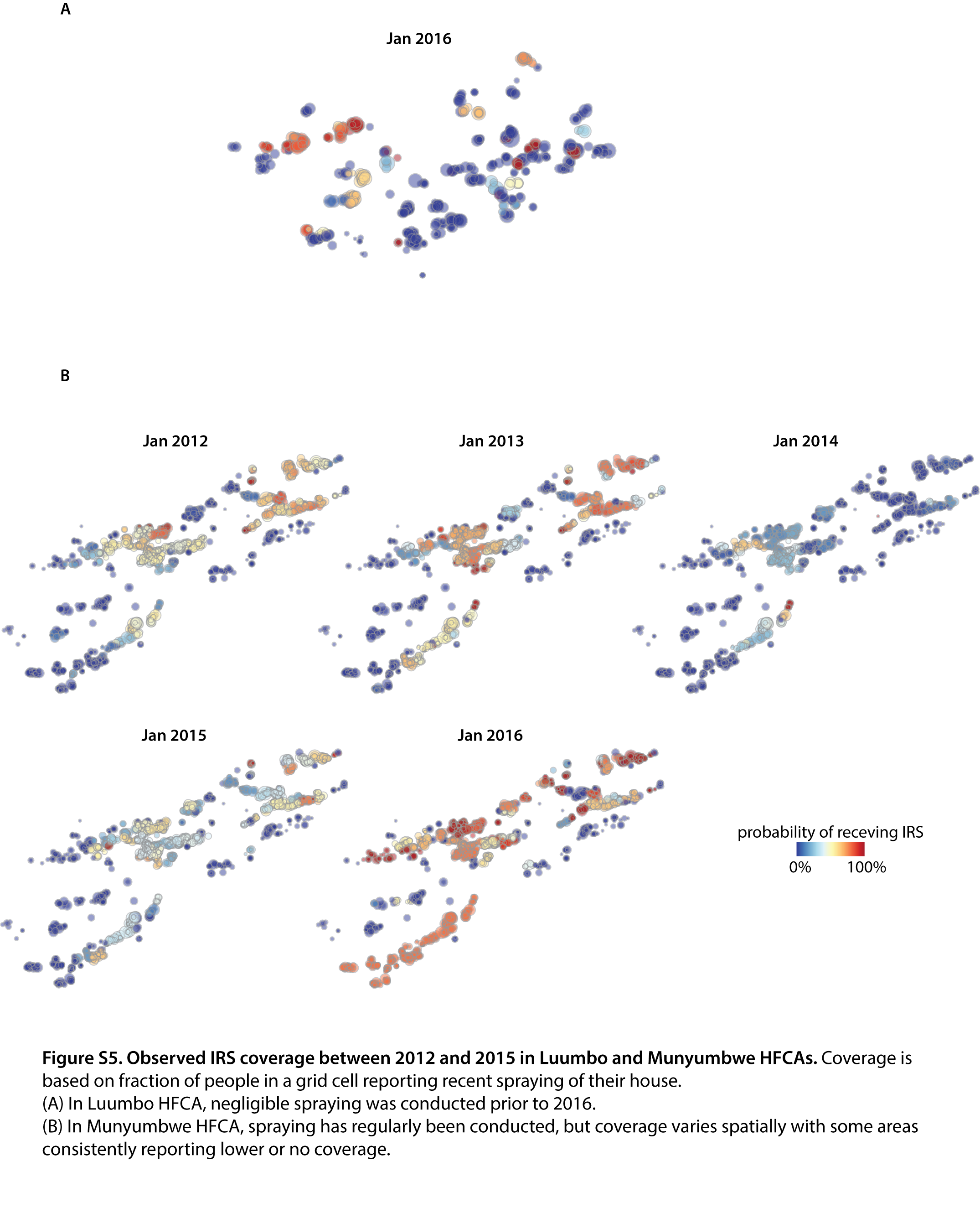

Supplement: Supplementary file 6 — Additional file 6. Observed IRS coverage between 2012 and 2015 in Luumbo and Munyumbwe HFCAs. [file 12936_2017_1903_MOESM6_ESM.png]

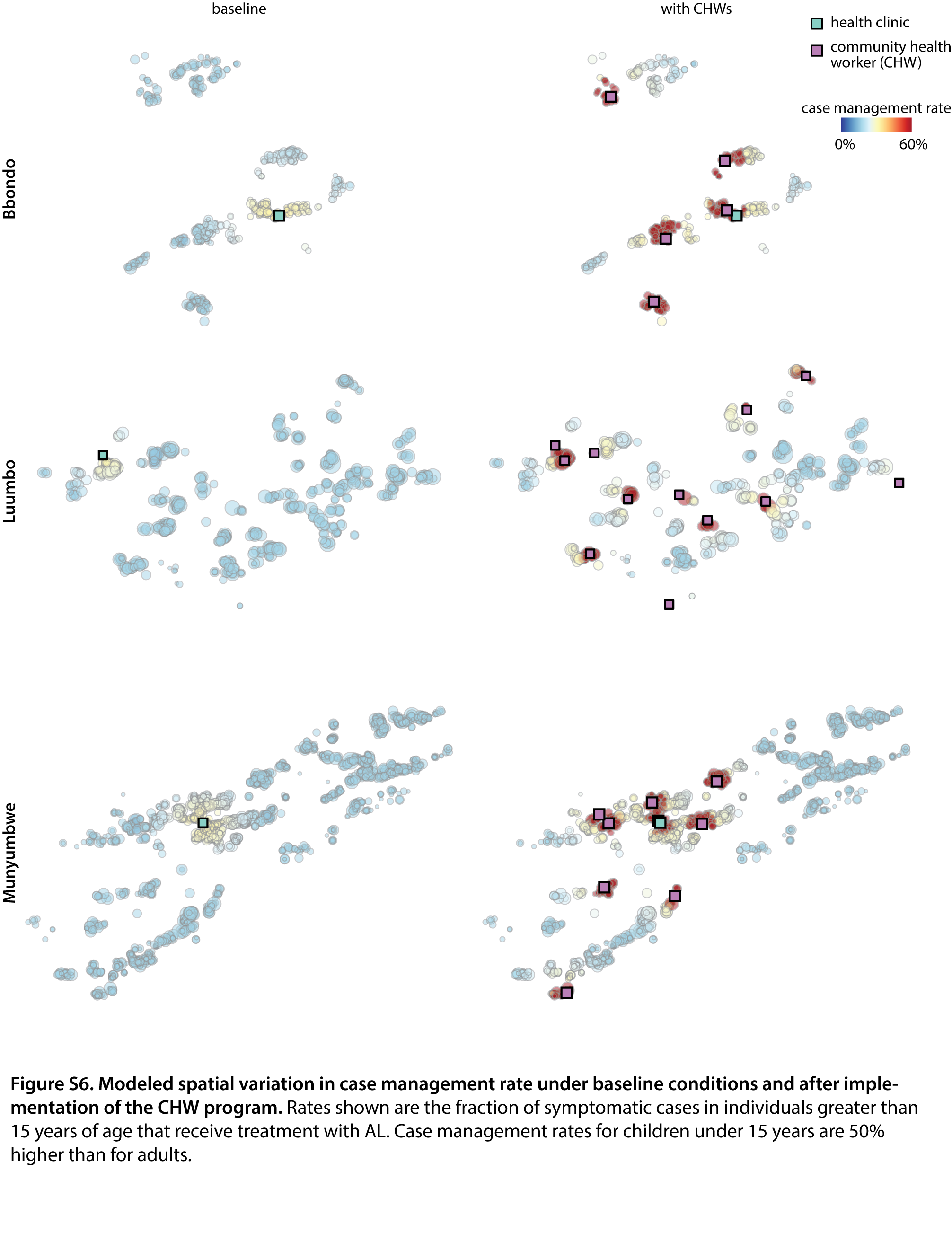

Supplement: Supplementary file 7 — Additional file 7. Modelled spatial variation in case management rate under baseline conditions and after implementation of the CHW programme. [file 12936_2017_1903_MOESM7_ESM.png]

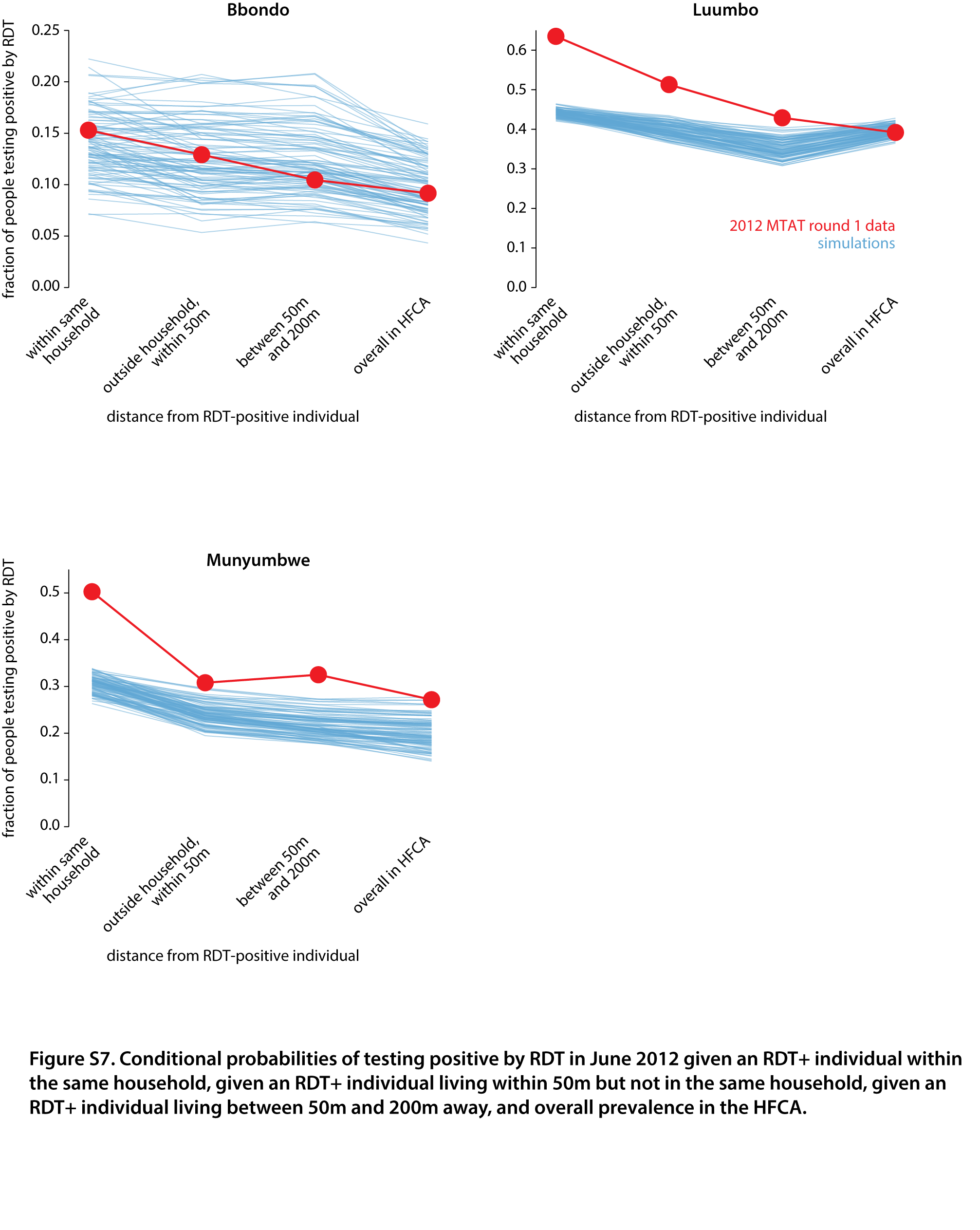

Supplement: Supplementary file 8 — Additional file 8. Conditional probabilities of testing positive by RDT in June 2012 given an RDT+ individual within the same household, given an RDT+ individual living within 50m but not in the same household, given an RDT+ individual living between 50m and 200m away, and overall prevalence in the HFCA. [file 12936_2017_1903_MOESM8_ESM.png]

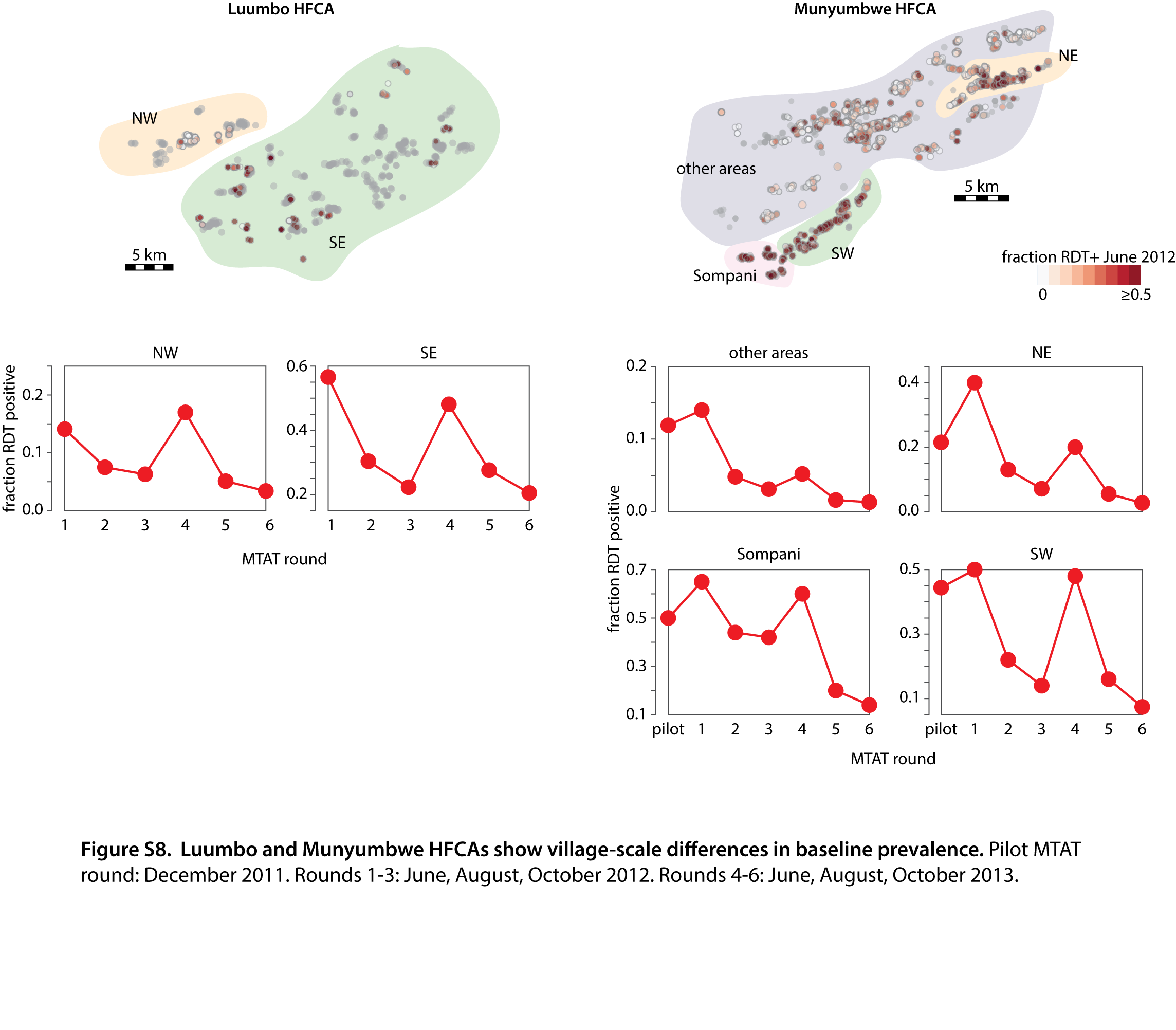

Supplement: Supplementary file 9 — Additional file 9. Luumbo and Munyumbwe HFCAs show village-scale differences in baseline prevalence. [file 12936_2017_1903_MOESM9_ESM.png]

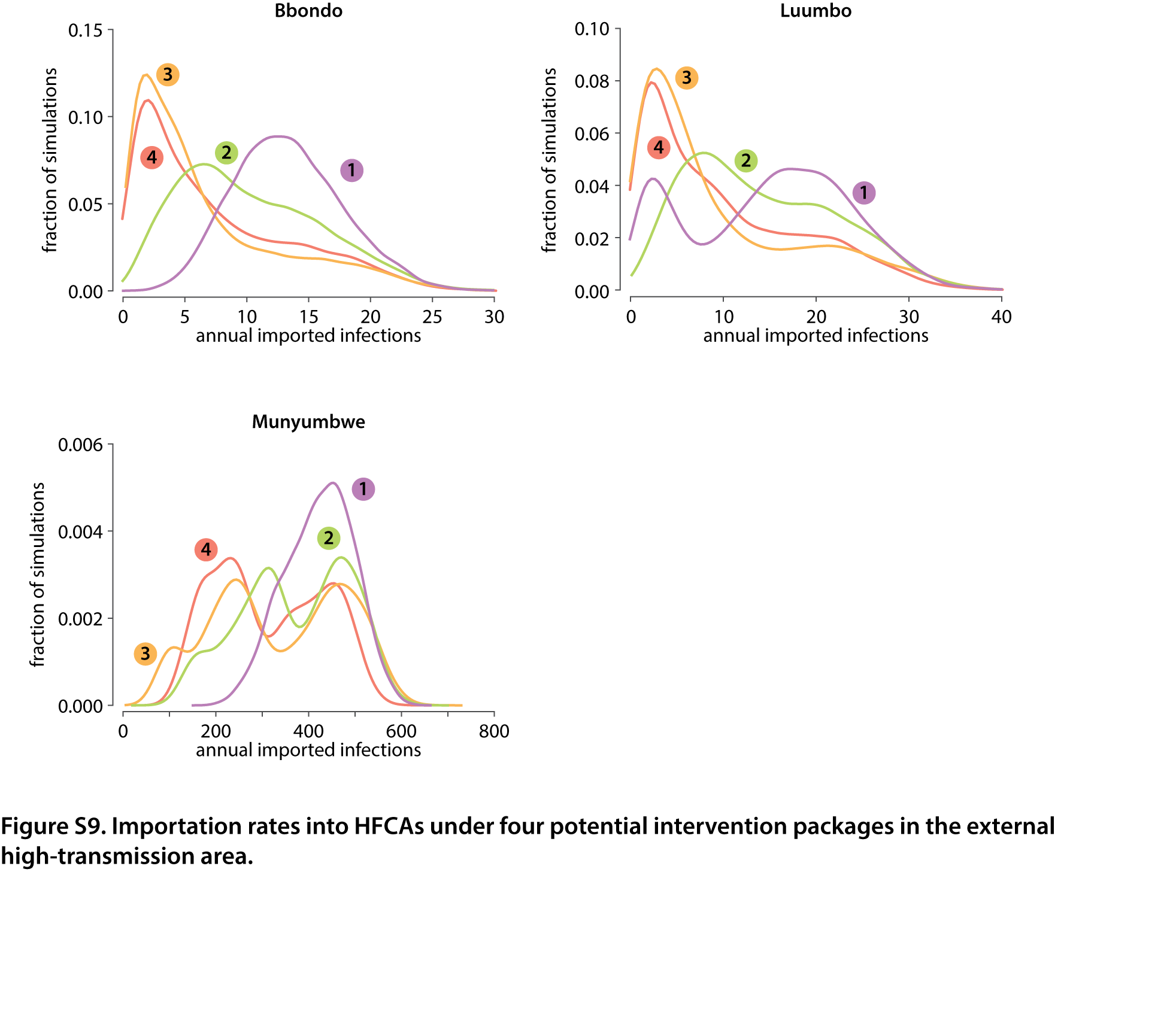

Supplement: Supplementary file 11 — Additional file 11. Importation rates into HFCAs under four potential intervention packages in the external high-transmission area. [file 12936_2017_1903_MOESM11_ESM.png]
